# Physical fitness of military policemen who practice CrossFit

**DOI:** 10.47626/1679-4435-2023-854

**Published:** 2023-04-18

**Authors:** Thiago Santos Evangelista, Giancarla Aparecida Botelho Santos

**Affiliations:** 1 Departamento de Medicina, Faculdade de Ciências da Saúde, Universidade Federal de Lavras, Lavras, MG, Brazil

**Keywords:** police, physical fitness, exercise, range of motion, articular, muscle strength, polícia, aptidão física, exercício, amplitude de movimento articular, força muscular

## Abstract

**Introduction:**

The practice of physical activity is directly related to the maintenance of health. Thus, the individual who is in the habit of practicing and who is well conditioned is able to perform various day-to-day functions with the least possible effort. In addition, good physical fitness is a requirement made for professionals of different categories, such as members of the security forces. Within this context, the military police officer must be within the standards of physical activity in order to exercise their ostensive functions. CrossFit is a training method that uses high intensity functional movements and aims to improve the physical form and health of the practitioner, thereby influencing its physical capacities.

**Objectives:**

To assess the physical fitness of military police officers who practice CrossFit.

**Methods:**

The sample consisted of 16 male active military police officers, practitioners of the institutional physical exercises, divided between CrossFit practitioners for at least 5 months (n = 10) and non-practitioners of extra-institutional exercises (n = 6). The following parameters were evaluated: level of physical activity, body mass index, fat percentage, flexibility, upper limb strength, and cardiorespiratory capacity.

**Results:**

The practice of CrossFit complementary to military physical training increased the values of upper limb strength, flexibility, and cardiorespiratory capacity among the components of physical fitness that were evaluated.

**Conclusions:**

The regular practice of CrossFit by military police suggests positive interference in some of the components of physical fitness and in the balance in strength gain, however more studies need to be conducted to investigate the significance of this effect.

## Abstract (pt)

**Introdução:**

A prática de atividade física está diretamente relacionada com a manutenção da saúde. Sendo assim, o indivíduo que tem o hábito de praticar e que é bem condicionado é capaz de executar várias funções cotidianas com menos esforço. Além disso, uma boa aptidão física é uma exigência feita a profissionais de diversas categorias, como os membros das forças de segurança. Dentro desse contexto, o policial militar precisa estar dentro dos padrões de atividade física para exercer suas funções ostensivas. O *crossfit* é um método de treinamento que utiliza movimentos funcionais em alta intensidade e que tem por objetivo melhorar a forma física e a saúde do praticante, influenciando, dessa forma, a aptidão física.

**Objetivos:**

Avaliar a aptidão física de policiais militares praticantes de *crossfit*.

**Métodos:**

A amostra foi composta por 16 policiais do sexo masculino, praticantes de atividade física institucional, divididos entre praticantes de *crossfit* por no mínimo 5 meses (n = 10) e não praticantes de atividade extrainstitucional (n = 6). Foram avaliados nível de atividade física, índice de massa corporal, percentual de gordura, flexibilidade, força de membros superiores e capacidade cardiorrespiratória.

**Resultados:**

A prática de *crossfit* complementar ao treinamento físico militar aumentou os valores de força de membros superiores, a flexibilidade e a capacidade cardiorrespiratória entre os componentes de aptidão física avaliados.

**Conclusões:**

A prática regular de *crossfit* por policiais militares parece interferir positivamente em alguns dos componentes da aptidão física e no equilíbrio no ganho de força; porém, mais estudos precisam ser realizados para investigar a significância desse efeito.

## INTRODUCTION

Physical activity can be characterized as any musculoskeletal movement that leads to energy expenditure. When body movements are structured and programmed repeatedly for a specific purpose, they are called physical exercises, which, besides helping to preserve health, also have the objective of improving physical conditioning. A well-conditioned individual has more strength, motor coordination, balance, agility, power, and reaction time to stimuli.^1^

Physical exercise is related to a higher quality of life and improvement in functional capacity, physical, social, and emotional aspects, general health status, vitality, and mental health.^2^ Moreover, it is known that physical conditioning plays a role in better work performance through its potential effects on reducing fatigue^3^ and protecting lower back musculoskeletal pain.^4^

Using several existing activities, including calisthenics and gymnastics, CrossFit institutionalizes these practices to create “complete athletes.”^5^ It was observed that the practice of CrossFit promotes an improvement in global physical capacity, reflecting positively in the parameters that define physical fitness, besides bringing benefits for the control of the lipid profile and body composition.^6^

The military career requires high physical fitness from the professional because in the exercise of their profession, the policeman is often exposed to violent and life-threatening situations.^7^ Based on this, the military policeman needs to be prepared to face the adversities of the profession, such as persecutions, escalades, fast movements, and even shootings. Consequently, lower-than-expected physical levels can reduce the military policeman’s performance concerning their professional needs, besides causing risks to their health.^8^

This way, we aimed to evaluate if military police officers who practice CrossFit have better physical fitness compared to those who perform only the conventional physical training required by the institution.

## METHODS

The sample was composed of 16 active military police officers, men, aged between 25 and 47 years, belonging to a battalion in the Brazilian state of Minas Gerais. They were divided into two groups: CrossFitters (n = 10) and non- CrossFitters (n = 6). As inclusion criteria, we had the following requirements: all military policemen should be in good health and practice the physical activity required weekly by the institution. In addition, the individuals in the CrossFit group should have practiced it for at least 5 months. Of the non-practicing individuals, none of them could perform physical activity outside of that required by the institution.

The level of physical activity of the military policemen was assessed by the International Physical Activity Questionnaire (IPAQ), in its short version, validated in Brazil.^9^ The questionnaire was filled out individually and with the help of the researcher, when necessary. Body mass index (BMI) was calculated by dividing body weight (kg) by the square of height (cm),^10^ using a portable stadiometer and a digital scale, both from Sanny. The fat percentage was determined according to previous protocols,^11^ using three skinfolds (chest, abdominal, and thigh) measured by a Cescorf skinfold caliper. The aerobic capacity was assessed by the Cooper test, in which the research participants were asked to run for 12 minutes without interruption on a running track inside the institution. During this time, the total distance traveled was recorded and then the maximum oxygen volume (VO_2max_) was calculated.^12^ Muscle strength was measured by the handgrip test with a JAMAR hydraulic dynamometer. The average of three measurements was taken.^13^ A Sanny Wells bench was used to determine the flexibility of the participants, and the values adopted as reference were collected from classical literature parameters.^14^

For data normalization, the Shapiro-Wilk test was used, observing that the data followed normal distribution, except for manual pressure (right arm), and Levene’s test for equality of variances. The t-test was used for independent samples and the Mann-Whitney test for comparison between groups. For all data, a significance level of p < 0.05 was adopted. The data were analyzed in the SPSS®, version 20.

All participants signed a free and informed consent form (FIC) after explanation of the research and the tests that would be performed. The project was approved by the Research Ethics Committee of the Universidade Federal de Lavras, under protocol number 84735318.8.0000.5148.

## RESULTS

After the analysis of the IPAQ short version questionnaires, it was found that none of the military police officers was classified as sedentary, regardless of the group. Of the 16 participants, 4 were classified as very active, 4 as active, and 8 as irregularly active. It is worth mentioning that 60% of the CrossFitters group was classified as very active or active, while in the non-CrossFitters group this percentage was only 33% (Table 1).

**Table 1:** Level of physical activity of military policemen among CrossFitters (n = 10) and non-CrossFitters (n = 6), according to the International Physical Activity Questionnaire (IPAQ) short version

Level of physical activity	Non-CrossFitters		CrossFitters	
	n	%	n	%
Very active	0	0	4	40
Active	2	33	2	20
Irregularly active A	3	50	3	30
Irregularly active B	1	17	1	10
Sedentary	0	0	0	0

The BMI analysis showed that the average mass of both groups was above the recommended mass, and they were classified as overweight.^15^ When comparing the groups, no statistically significant difference was found. The BMI values obtained were 26.34±2.55 kg/m^2^ for the non-practicing group and 25.11±1.62 kg/m^2^ for the CrossFit group (Table 2). It is worth mentioning that, according to some authors, the BMI measurement is not very reliable, because it cannot distinguish bone mass from muscle mass and from excess fat.^16^

**Table 2:** Values of physical fitness variables of military policemen who practice CrossFit (n = 10) and who do not (n = 6)

Fitness variables	Non-CrossFitters	CrossFitters
BMI (kg/m^2^)	26.34±2.55	25.11±1.62
% fat	15.18±3.47	12.30±2.53
Flexibility (cm)	14.66±6.11	24.50±1.62
LUL strength (kg)	43.06±6.45	51.86±5.77
RUL strength (kg)	48.08±4.57	50.36±1.90
VO^2^max (mL.kg.min^-1^)	42.28±2.35	46.18±6.62

Student’s t test: p ≤ 0.05.

BMI= body mass index; RUL = right upper limb; LUL = left upper limb; VO_2max_ = maximal oxygen volume.

Regarding the fat percentage, in the non-CrossFitters group it was 15.18 ± 3.47, and in the CrossFitters group, 12.3 ± 2.53 (Table 2). Although no statistically significant difference was observed, the percentage found in the group of non-CrossFitters was higher if compared to the other group, indicating a higher fat mass in military policemen who only do the standard military physical training.

In the flexibility analysis, the group of military police officers who practiced CrossFit showed flexibility considered average. The group of non-practitioners had a flexibility considered bad. The flexibility of the non-practicing group was 14.66±6.11 cm and the practicing group, 24.5±1.62 cm (Table 2). These data suggest a positive interference of the practice of CrossFit in this aspect, although, when comparing the values between the groups, no statistically significant difference was observed.

As for muscular fitness, it was observed that the strength of both upper limbs was equivalent between the groups. The left upper limb strength of the non-practicing group was 43.06±6.45 kg, while that of the practicing group was 51.86±5.77 kg. In the right upper limb, the handgrip strength was 48.08±4.57 kg and 50.36±1.90 kg (Table 2), respectively.

As for the cardiorespiratory capacity of the military policemen, it was observed that both groups obtained VO_2max_ averages considered excellent, with the group of CrossFitters presenting slightly higher averages. The VO_2max_ of the non-practicing group was 42.28±2.35 mL.kg.min^-1^, while the practitioners group was 46.18±6.62 mL.kg.min^-1^ (Table 2).

## DISCUSSION

Despite its widespread practice, CrossFit’s benefits have yet to be been fully evidenced in the literature.^17^ Therefore, the present study aimed to evaluate if the practice of this sport modality by military policemen improves their physical fitness compared to policemen who practice only the usual military physical training. Therefore, we performed anthropometric, flexibility, strength, and cardiorespiratory capacity measurements.

According to the literature,^18,19^ CrossFit training sessions include high-intensity exercises, executed quickly, repetitively, and with little or no interval between sets, which may, in excess and/or inadequate execution, lead to different patterns of injuries. One study, when comparing CrossFit training to the recommendations of the American College of Sports Medicine (ACSM), described it as a strenuous and very difficult activity.^20^ Furthermore, similar oxidative stress is seen between CrossFit and running on a treadmill at an intensity of at least 90% of the maximum heart rate.^21^ Thus, those who practice CrossFit get used to high-intensity exercises.

The IPAQ questionnaire, when assessing the level of physical activity, weights the intensity at which the exercise is performed, considering the most exhausting activities with the greatest weight.^9^ This fact, added to the high intensity in which the practice of CrossFit is performed, is one of the possible justifications for the higher level of physical activity found in military police officers practicing this sport in the present study.

A reduction in anthropometric measurements among CrossFit practitioners has been previously demonstrated.^22^ However, no significant changes were found in body composition after 8 weeks of practice.^23^ On the other hand, an increase in lean body mass and a reduction in fat percentage were observed, especially in women, after 3 months of CrossFit training.^24^ In the present study, BMI and fat percentage were similar in both groups, suggesting that other variables besides physical activity should be investigated. Food intake, sleep quality, and environmental conditions may interfere with body weight and fat percentage,^25^ and also with the number and duration of training sessions per week.^24^ The control of these variables would be an important step to understanding the role of CrossFit in the anthropometric measurements of the population studied, whose maintenance of eutrophy is essential for a good professional career.

When evaluating the flexibility of military police officers, percentages of flexibility below the average were found.^26^ According to the authors, a possible explanation would be the dispersion of the data. In the present study, the military police officers who did not practice CrossFit showed poor flexibility, while the group of practitioners showed average flexibility. Thus, it can be observed that the flexibility of military police officers practicing CrossFit was higher than that of non-practitioners, suggesting a positive influence of the practice of this sport on physical fitness. Furthermore, some authors infer that, because CrossFit uses elements from gymnastics and Olympic weight lifting, which demand a lot from the range of movement, there may be interference in flexibility.^27^

Regarding upper limb strength, the military policemen practicing CrossFit showed strength similar to those who did not practice it, indicating that the additional training of this sport modality was not enough to promote an increase in this physical fitness valence. However, both groups of military police officers showed higher upper limb strength than that found in previous studies.^28^ This fact may indicate the demands of the profession itself interfering with upper limb strength, since military physical training includes fights, shooting practice, and arm flexion, among other exercises, demanding greater strength both in the upper limbs and in general. Furthermore, it is important to point out that when comparing the strength of the right and left upper limbs within the same group, it is observed that the practice of CrossFit minimized the existing strength differences, suggesting the potential of the activity in the symmetrical gain of strength. For military police officers, who use heavy equipment such as belts, holsters, and bulletproof vests during their working hours, greater upper limb strength is important, because it reduces the probability of occurrence of injuries and musculoskeletal pain.^13^

Regarding VO_2max_, both the group that did not practice CrossFit and the group that practiced it showed excellent cardiorespiratory capacity. Military police officers are required to regularly practice aerobic physical exercises, which usually consist of walking or running at an intensity of approximately 60% of maximum HR.^7^ Exercises performed at this intensity are capable of increasing the levels of the cardiorespiratory component, justifying the similar results between the groups.^29^

However, the addition of CrossFit training two to three times a week was not efficient to increase the VO_2max_ in relation to the non-practitioner group, although CrossFit practice has highly varied physiological demands, leading to parameters such as HR between 54 and 98% of HR _max_, blood lactate levels between 6 and 15 mmol/L and VO_2max_ between 57 and 66%.^17^ A recent research, when comparing CrossFit practitioners with bodybuilding practitioners individually observed a significant difference in VO2max, with CrossFit practitioners presenting higher values of this variable.^30^ However, when comparing the mean, corroborating the results obtained in this study, no significant differences were observed. However, another study,^8^ when analyzing 43 healthy adults with different types of physical conditioning, reported an improvement in VO_2max_ after 10 weeks of CrossFit practice. This increase occurred regardless of sex and fitness level. On the other hand, another study found an improvement in VO_2max_ after 3 months of practice, especially among women.^24^

Thus, based on this research, it is not possible to state that the introduction of CrossFit into military physical training would improve the VO_2max_ of military police officers. Studies are needed to investigate both military physical training and CrossFit training, evaluating intensity, frequency, and the way they are performed, as well as the individual’s lifestyle.

The results of the present study allowed the evaluation of the components of physical fitness of military police officers and the interference of the practice of CrossFit on these components. It was observed that the regular practice of this sport modality did not interfere significantly with the physical fitness of the population, although the CrossFit practitioners showed slightly higher upper limb strength and cardiorespiratory capacity. Thus, future investigations involving other variables and encompassing larger samples are essential to evaluate the significant effects of this practice. We suggest that the introduction to CrossFit movements in military physical training performed at high intensities and under supervision may positively influence the performance of military police officers’ work activities.

## CONCLUSIONS

Military policemen showed a good level of physical activity and good components of physical fitness, suggesting good overall health status. However, some variables need to be improved for a better quality of life and work, such as BMI and flexibility. Although no significant differences were observed in the variables evaluated, the results indicate that the practice of CrossFit can positively interfere in the execution of the military policemen’s work activities when done systematically and under supervision. New studies controlling other variables, such as military physical training, nutrition, adherence to physical exercises outside the work environment, comorbidities, and environmental conditions, should be conducted for a better understanding of the interference of CrossFit in the physical conditioning of military police officers.

## References

[1] Nancy A, Dasso MSN (2019). How is exercise different from physical activity? A concept
analysis. Nurs Forum.

[2] Mandolesi L, Polverino A, Montuori S, Foti F, Ferraioli G, Sorrentino P (2018). Effects of physical exercise on cognitive functioning and
wellbeing: biological and psychological benefits. Front Psychol.

[3] Song B, Yang Y, Bai W, Li Z, Wan J, Teng X (2019). Effect of physical exercise on young anesthesiologists with
on-call-related fatigue. Psychol Health Med.

[4] Vanti C, Andreatta S, Borghi S, Guccione AA, Pillastrini P, Bertozzi L (2019). The effectiveness of walking versus exercise on pain and function
in chronic low back pain: a systematic review and meta-analysis of
randomized trials. Disabil Rehabil.

[5] Rojo JR, Quitzau EA, Santos ACB, Moraes e (2019). Silva M. “Nada se cria...”: o crossfit enquanto prática
corporal ressignificada. Rev Motriviv.

[6] Dehghanzadeh Suraki R, Mohsenzade M, Tibana RA, Ahmadizad S (2021). Effects of CrossFit training on lipid profiles, body composition
and physical fitness in overweight men. Sport Sci Health.

[7] Lima-Dos-Santos AL, Domingos-Gomes JR, Dantas Andrade OS, Cirilo-Sousa MDS, Domingos da Silva Freitas E, Gomes Silva JC (2018). Health-related physical fitness of military police officers in
Paraiba, Brazil. Rev Bras Med Trab.

[8] Martins RC, Ramos MFH, Silva EP, Pereira ECDCS (2020). Lesões musculoesqueléticas em Policiais Militares:
uma revisão da literatura. Res Soc Dev.

[9] Matsudo S, Araújo T, Matsudo V, Andrade D, Andrade E, Oliveira LC (2001). Questionário internacional de atividade física
(IPAQ): estudo de validade e reprodutibilidade no Brasil. Ativ Fís Saúde.

[10] Booth ML, Hunter C, Gore CJ, Bauman A, Owen N (2000). The relationship between body mass index and waist circumference:
implications for estimates of the population prevalence of
overweight. Int J Obes Relat Metab Disord.

[11] Jackson AS, Pollock ML (1978). Generalized equations for predicting body density of
men. Br J Nutr.

[12] Cooper KH (1968). A means of assessing maximal oxygen intake. Correlation between
field and treadmill testing. JAMA.

[13] Fernandes ADA, Marins JCB (2011). Teste de força de preensão manual: análise
metodológica e dados normativos em atletas. Fisioter. Mov.

[14] Wells KF, Dillon EK (1952). The sit and reach-a test of back and leg
flexibility. Res Q.

[15] World Health Organization (WHO) (1995). Physical status: The use and interpretation of anthropometry.

[16] Rezende FAC, Rosado LEFPL, Franceschinni SDCC, Rosado GP, Ribeiro RDCL (2010). Aplicabilidade do índice de massa corporal na
avaliação da gordura corporal. Rev Bras Med Esport.

[17] Claudino JG, Gabbett TJ, Bourgeois F, Souza HS, Miranda RC, Mezencio B (2018). CrossFit overview: systematic review and
meta-analysis. Sports Med Open.

[18] Dominski FH, Siqueira TC, Serafim TT, Andrade A (2018). Perfil de lesões em praticantes de CrossFit:
revisão sistemática. Fisioter Pesqui.

[19] Minghelli B, Vicente P (2019). Musculoskeletal injuries in Portuguese CrossFit
practitioners. J Sports Med Phys Fitness.

[20] Drum SN, Bellovary BN, Jensen RL, Moore MT, Donath L (2017). Perceived demands and postexercise physical dysfunction in
CrossFit(R) compared to an ACSM based training session. J Sports Med Phys Fitness.

[21] Kliszczewicz B, Quindry CJ, Blessing LD, Oliver DG, Esco RM, Taylor JK (2015). Acute exercise and oxidative stress: CrossFit() vs. Treadmill
Bout. J Hum Kinet.

[22] Guerro PRS, Fachineto S (2016). Efeitos de um programa de treinamento crossfit sobre
composição corporal, força muscular e
freqüência cardíaca de repouso em homens jovens adultos
treinados. EFDeportes.com.

[23] Heinrich KM, Patel PM, O’Neal JL, Heinrich BS (2014). High-intensity compared to moderate-intensity training for
exercise initiation, enjoyment, adherence, and intentions: an intervention
study. BMC Public Health.

[24] Murawska-Cialowicz E, Wojna J, Zuwala-Jagiello J (2015). Crossfit training changes brain-derived neurotrophic factor and
irisin levels at rest, after wingate and progressive tests, and improves
aerobic capacity and body composition of young physically active men and
women. J Physiol Pharmacol.

[25] Imamura H, Shibuya S, Uchida K, Teshima K, Masuda R, Miyamoto N (2004). Effect of moderate exercise on excess post-exercise oxygen
consumption and catecholamines in young women. J Sports Med Phys Fitness.

[26] Domingos-Gomes JR, Oliota-Ribeiro LS, Silva JDS, Melo ACD, Albuquerque Neto SLD, Cirilo-Sousa MDS (2016). Comparação da aptidão física
relacionada à saúde e sua associação com o tempo
de serviço entre policiais militares de operações
especiais e de trânsito. J Physic Educ.

[27] Tibana RA, Sousa NMF, Barros GC, Prestes J (2017). Correlação das variáveis
antropométricas e fisiológicas com o desempenho no
Crossfit®. RBPFEX.

[28] Dantas TSP (2018). Análise da hipotensão, força, potência e
temperatura corporal após sessão de Crossfit®.

[29] Garber CE, Blissmer B, Deschenes MR, Franklin BA, Lamonte MJ, Lee IM (2011). American College of Sports Medicine position stand. Quantity and
quality of exercise for developing and maintaining cardiorespiratory,
musculoskeletal, and neuromotor fitness in apparently healthy adults:
guidance for prescribing exercise. Med Sci Sports Exerc.

[30] Pires PJSN (2018). Análise metabólica do Crossfit®: resposta
energética dos diferentes benchmarks (WOD’S).

